# miR-1238 inhibits cell proliferation by targeting LHX2 in non-small cell lung cancer

**DOI:** 10.18632/oncotarget.4232

**Published:** 2015-05-22

**Authors:** Xiangguang Shi, Lei Zhan, Can Xiao, Zhe Lei, Haiping Yang, Longqiang Wang, Jun Zhao, Hong-Tao Zhang

**Affiliations:** ^1^ Soochow University Laboratory of Cancer Molecular Genetics, Medical College of Soochow University, Suzhou, China; ^2^ Suzhou Key Laboratory for Molecular Cancer Genetics, Suzhou, China; ^3^ The First Affiliated Hospital of Soochow University, Medical College of Soochow University, Suzhou, China

**Keywords:** NSCLC, miR-1238, LHX2, cell proliferation

## Abstract

In human cancers, dysregulated expression of LIM-homeobox gene 2 (LHX2) and downregulation of miR-1238 has been reported separately. However, the relationship between them remains unclear. We investigated the functional contribution of miR-1238 to the regulation of *LHX2* in non-small cell lung cancer (NSCLC). Here, computational algorithms predicted that the 3′-untranslated region (3′-UTR) of *LHX2* is a target of miR-1238. Luciferase assays validated that miR-1238 directly bound to 3′-UTR of *LHX2*. qRT-PCR and western blot analyses further confirmed that overexpression of miR-1238 mimic in NSCLC A549 and LTEP-α-2 cells inhibited endogenous expression of LHX2 mRNA and protein. Moreover, ectopic expression of miR-1238 in NSCLC A549 and LTEP-α-2 cells suppressed cellular viability and proliferation. siRNA-induced knockdown of LHX2 copied the phenotype of miR-1238 overexpression in NSCLC A549 and LTEP-α-2 cells and LHX2 knockdown inhibited cell cycle. In addition, miR-1238 expression was frequently decreased in human NSCLC tissues and reversely correlated with *LHX2* expression, which was increased in NSCLC tissues. Collectively, our findings demonstrate that miR-1238 inhibit the proliferation of NSCLC cells at least partly via repression of *LHX2*, shedding light on the mechanistic interaction of miR-1238 and *LHX2* in NSCLC carcinogenesis. Furthermore, our data suggest that expression of miR-1238 could be a promising therapeutic strategy for NSCLC treatment.

## INTRODUCTION

Lung cancer is the leading cause of cancer deaths in men and women worldwide [1]. Among all lung cancer cases, non-small cell lung cancer (NSCLC) accounts for approximately 85% [2]. Despite improvement in cancer therapy, the prognosis for NSCLC patients remains poor, with a 5-year survival rate of approximately 10% [3]. Thus, uncovering the mechanisms underlying NSCLC carcinogenesis is crucial.

LIM-homeobox gene 2 (LHX2), a member of the LIM homeobox family of proteins, is implicated in the control of cell differentiation and proliferation [4] and plays an important role in embryogenesis [5]. In addition to the well-established roles of LHX2 in physiological conditions, LHX2 is involved in various human cancers. For instance, Kuzmanov *et al*. reported that LHX2 promoted tumor growth and metastasis by inducing platelet-derived growth factor (PDGF)-B signaling in breast cancer cells [6]. Although high levels of LHX-2 expression were found in chronic myelogenous leukaemia (CML) [7] and a variety of human solid tumors, including gastrointestinal cancer, pancreatic cancer and kidney cancer [6, 8], the expression and regulation of *LHX2* in NSCLC carcinogenesis has not yet been defined. We here report the high expression of *LHX2* in human NSCLC cells and tissues. To explore the mechanisms underlying upregulation of *LHX2* in NSCLC, we focused on the role of microRNA (miRNA) in the expression of *LHX2*.

MiRNAs are small noncoding RNAs of 19-24 nucleotides in length, which act as the crucial post-transcriptional regulators of gene expression by targeting mRNAs [9-11]. MiRNAs sequences are distributed throughout the human genome [12] and regulate more than 30% of protein-coding genes [13]. Therefore, miRNAs participate in many biological processes, including development, cell growth, differentiation, migration and carcinogenesis [14-16]. More recently, Balaguer *et al*. [17] and Lin *et al*. [18] reported that miR-1238 was down-regulated in tumor tissues from patients with hereditary nonpolyposis colorectal cancer (HNPCC) and cervical cancer, respectively. Takikawa *et al*. also found the reduced expression of miR-1238 in pancreatic cancer Panc-1 cells [19]. These findings suggested that miR-1238 is associated with human cancers. However, no evidence of miR-1238 expression was identified in NSCLC. Besides, using computational algorithms, we predicted that miR-1238 can directly target the 3′-untranslated region (3′-UTR) of *LHX2*. This inspired us to focus on the functional contribution of miR-1238 to the upregulation of *LHX2* in NSCLC.

In the present study, for the first time, we examined the relationship between miR-1238 and *LHX2* expression, and explored the mechanistic role of miR-1238 in regulating the expression of *LHX2* in NSCLCs. We found that miR-1238 level was down-regulated in 62.0% (31/50) of NSCLC tissues, 24 of which (77.4%) showed up-regulated expression of *LHX2* mRNA. Moreover, cell-based and biochemical analyses revealed that miR-1238 diminished the expression of LHX2 by targeting *LHX2* which is required for NSCLC cell proliferation.

## RESULTS

### LHX2 expression is up-regulated in NSCLC cells and tissues

**Figure 1:**
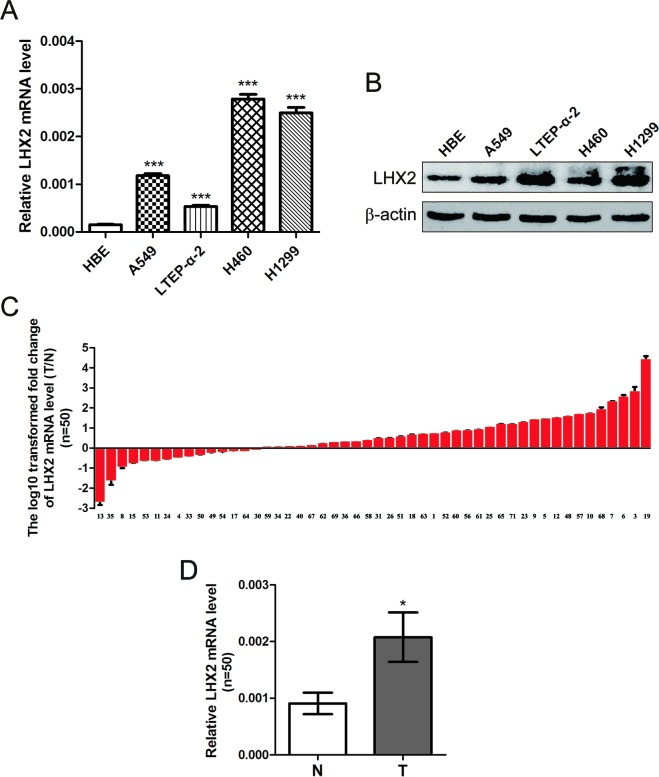
Expression of *LHX2* is up-regulated in human NSCLC cells and tissues **A.** qRT-PCR analysis of *LHX2* mRNA levels in HBE cells and NSCLC A549, LTEP-α-2, H460 and H1299 cells. *LHX2* mRNA levels are expressed as a relative index normalized to β-actin. **B.** Western blot analysis of LHX2 protein expression in HBE cells and NSCLC A549, LTEP-α-2, H460 and H1299 cells. β-actin was used as internal control. **C.** qRT-PCR analysis of relative *LHX2* mRNA levels in 50 NSCLC tissues (T) and paired noncancerous lung tissues (N). *Y*-axis represents the log_10_ transformed fold change of T/N mRNA expression ratio of *LHX2*. The number of each sample is indicated below *x*-axis. **D.** Difference in *LHX2* mRNA expression between T and N. *P < 0.05; ***P < 0.001.

LHX2 functions as a tumor promoter in breast cancer cells [6]. Nevertheless, little is still known about the role of LHX2 in NSCLC. To explore this, we first examined LHX2 expression in 4 NSCLC cell lines and 50 paired NSCLC tissues and adjacent cancer-free lung tissues. As shown in Figure 1A, *LHX2* mRNA levels were significantly higher in A549, LTEP-α-2, H460, and H1299 cells than HBE cells (*P* < 0.001, *P* < 0.001, *P* < 0.001, and *P* < 0.001, respectively). LHX2 protein levels were consistently obtained in 5 cell lines (Figure 1B). Moreover, among 50 randomly selected paired tissues from NSCLC patients, 35 tumors (70.0%) showed a significant increase in *LHX2* mRNA expression when compared with paired noncancerous lung tissues (*P* < 0.05; Supplemental Table S1, Figure 1C and 1D). The results suggested that LHX2 may play a tumor-promoting role in NSCLC.

### Expression of miR-1238 is reduced and reversely correlated with LHX2 level in NSCLC cells and tissues

**Figure 2:**
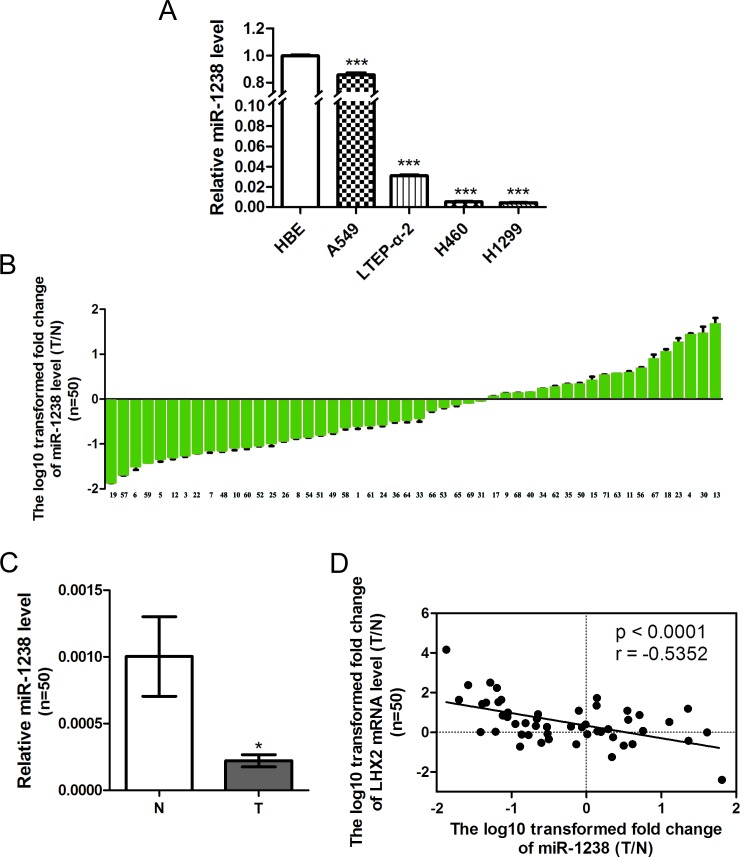
Level of miR-1238 is reduced in NSCLC cells and tissues and reversely correlated with *LHX2* expression in human NSCLC tissues **A.** MiR-1238 levels expressed in HBE cells and NSCLC A549, LTEP-α-2, H460 and H1299 cells. MiR-1238 level for HBE cells was assigned the value 1, and the relative miR-1238 level of NSCLC cells was recalculated accordingly. MiR-1238 levels are expressed as a relative index normalized against U6. **B.** Relative miR-1238 levels in 50 NSCLC tissues (T) and paired noncancerous lung tissues (N). *Y*-axis represents the log_10_ transformed fold change of miR-1238 expression ratio (T/N). The number of each sample is indicated below *x*-axis. **C.** Difference in miR-1238 level between T and N. **D.** Correlation between miR-1238 level and *LHX2* mRNA expression in 50 paired NSCLC tissues. MiR-1238 and *LHX2* mRNA levels are expressed as relative index normalized against U6 and β-actin, respectively. *X* and *y* axes represent the log_10_ transformed fold change of T/N mRNA expression ratios of miR-1238 and *LHX2*, respectively. *P < 0.05; ***P < 0.001.

As illustrated in Figure 2A, miR-1238 expression level was significantly lower in A549, LTEP-α-2, H460, and H1299 cells than HBE cells (*P* < 0.001, *P* < 0.001, *P* < 0.001, and *P* < 0.001, respectively). Furthermore, among 50 randomly selected paired tissues from NSCLC patients, 31 tumors (62.0%) showed a significant reduction in miR-1238 level when compared with paired noncancerous lung tissues (Supplemental Table S1, Figure 2B and 2C; *P* < 0.05). No significant difference in miR-1238 level or *LHX2* mRNA was observed between NSCLCs when classified by various clinicopathologic characteristics (Supplemental Table S2). Importantly, the ratio of miR-1238 level (T/N) was inversely correlated with that of *LHX2* mRNA level (T/N) in 50 paired tissues (*P* < 0.0001; Figure 2D). Of 31 NSCLC tissues with low miR-1238 level, 24 tumors (77.4%) showed high expression of *LHX2* mRNA (Figure 2D), suggesting a regulatory role of miR-1238 in *LHX2* expression in NSCLCs.

### miR-1238 reduces LHX2 expression by targeting LHX2 3′-UTR in NSCLC cells

**Figure 3:**
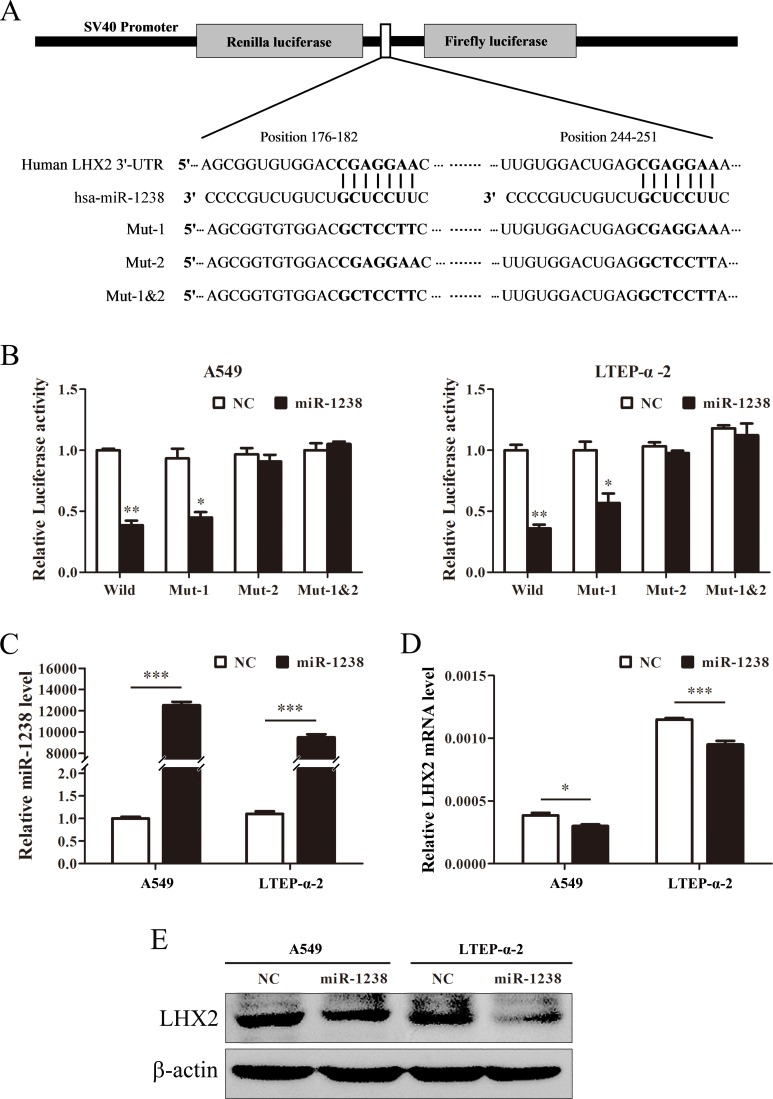
miR-1238 reduces *LHX2* expression by directly targeting *LHX2* 3′-UTR **A.** Schematic graph showing the subcloning of the predicted miR-1238 binding sites (positions 176-182 and 244-251) of *LHX2* 3′-UTR into psiCHECK-2 luciferase construct. Predicted duplex formation between miR-1238 and wildtype/mutant of miR-1238 binding sites is indicated. **B.** Luciferase activities of the construct containing the wildtype or mutant *LHX2* 3′-UTR reporter gene in A549 and LTEP-α-2 cells cotransfected with negative control (NC) or miR-1238. Scrambled sequences were used as NC. Relative *Renilla* luciferase activity is determined followed by normalizing against the firefly luciferase activity. **C.** qRT-PCR analysis of miR-1238 levels in A549 and LTEP-α-2 cells transfected with miR-1238 mimics or NC for 72 h. U6 was used as internal control. **D.** *LHX2* mRNA expression in A549 and LTEP-α-2 cells transfected with miR-1238 mimics or NC. β-actin was used as internal control. **E.** LHX2 protein levels in A549 and LTEP-α-2 cells transfected with miR-1238 mimics or NC. *P < 0.05; **P < 0.01; ***P < 0.001.

Given the fact miRNAs can regulate various biological processes including cell proliferation by targeting proliferation-related genes [2], we used TargetScanHuman v6.2 (http://www.targetscan.org) to predict the targets of miR-1238. As predicted, the 3′-UTR of the mRNA encoding *LHX2* harbors two miR-1238 binding sites (positions 176-182 and 244-251 in the NM_004789 RefSeq transcript), suggesting that *LHX2* could be a potential target of miR-1238. To test this, we subcloned *LHX2* 3′-UTR containing the wildtype/mutants of the two miR-1238 target sites into psiCHECK-2 vector (Figure 3A) and cotransfected the luciferase construct with miR-1238 mimics into A549 and LTEP-α-2 cells. As illustrated in Figure 3B, miR-1238 significantly attenuated the luciferase activities in A549 and LTEP-α-2 cells transfected with the *LHX2* 3′-UTR wildtype or mutant-1 but did not inhibit the luciferase activities in A549 and LTEP-α-2 cells with the mutant-2 and mutant-1&2 constructs, suggesting that miR-1238 can selectively bind to the sequence (position 244-251) of *LHX2* 3′-UTR. Furthermore, overexpression of miR-1238 (Figure 3C) significantly diminished LHX2 mRNA and protein levels in A549 and LTEP-α-2 cells (Figure 3D and 3E). Taken together, the results suggested that miR-1238 can directly target the 3′-UTR of *LHX2*.

### miR-1238 overexpression inhibits NSCLC cell proliferation and knockdown of LHX2 represses NSCLC cell proliferation

**Figure 4:**
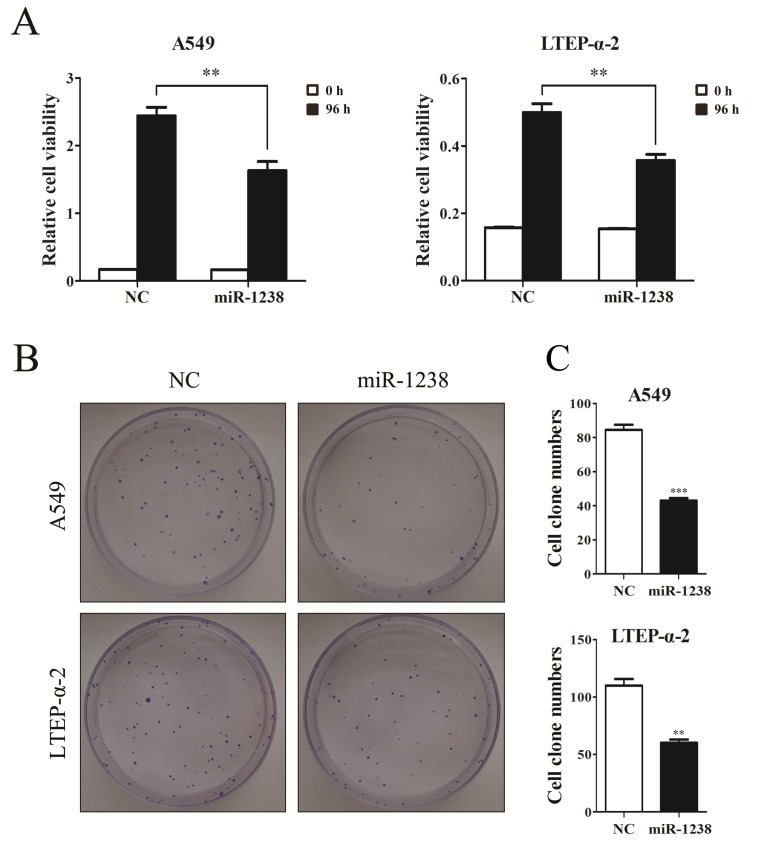
Overexpression of miR-1238 mimic inhibits NSCLC cell viability and proliferation **A.** CCK-8 assay of cell viability in A549 and LTEP-α-2 cells transfected with miR-1238 mimics or NC for 96 h. **B.** Representative images of clonogenic analysis for cell proliferation in A549 and LTEP-α-2 cells transfected with miR-1238 mimics or NC. **C.** Bar charts showing clonogenic growth of A549 and LTEP-α-2 cells transfected with miR-1238 mimics or NC. Values are represented as means±SE from three measurements. **P < 0.01; ***P < 0.001.

Considering our findings described above and the facts that miR-1238 was down-regulated in HNPCC and pancreatic cancer cells [17, 19], we hypothesized that miR-1238 may serve as a tumor suppressor in NSCLC. Thus, we overexpressed miR-1238 in NSCLC cells and then evaluated the role of miR-1238 in cell growth inhibition. CCK-8 assays showed that the proliferation ability of NSCLC cells overexpressing miR-1238 was significantly lower than that of control cells (*P* < 0.01; Figure 4A). The results were further confirmed by clonogenic assay in A549 and LTEP-α-2 cells (Figure 4B and 4C), suggesting that miR-1238 played an important role in repressing NSCLC cell proliferation.

**Figure 5:**
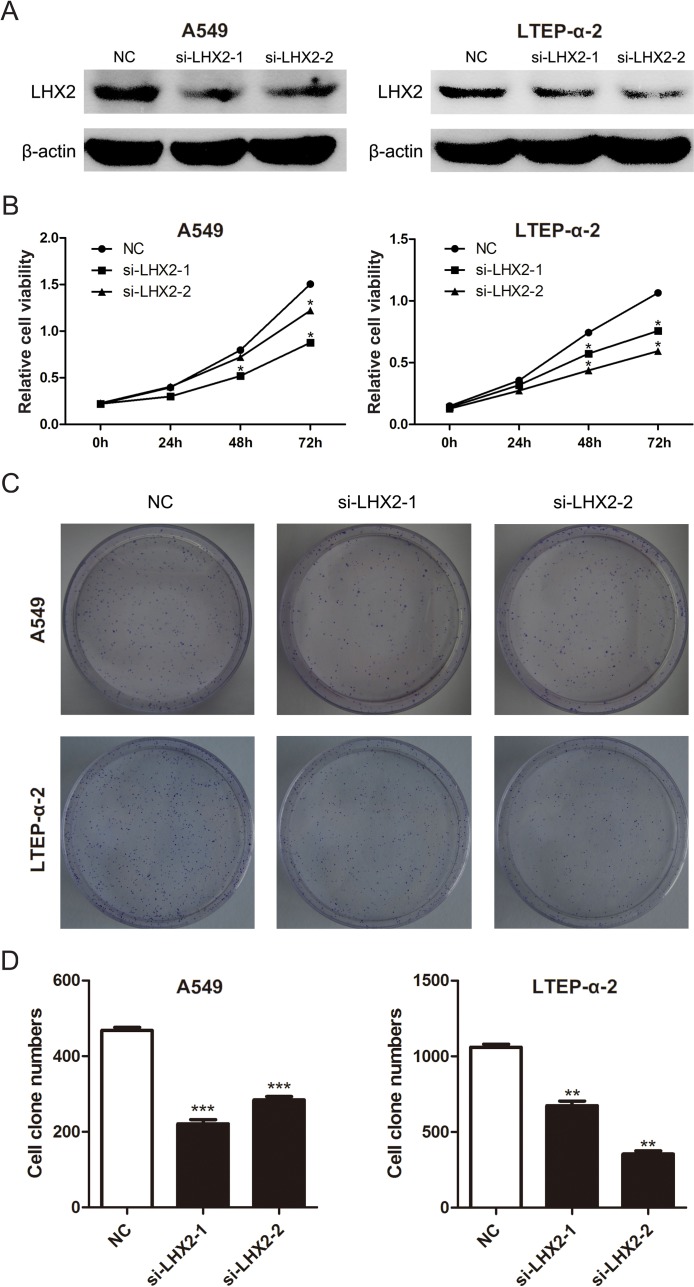
Knockdown of *LHX2* represses NSCLC cell viability and proliferation **A.** LHX2 protein levels in A549 and LTEP-α-2 cells transfected with NC, si-LHX2-1 and si-LHX2-2. **B.** CCK-8 assay of cell viability in A549 and LTEP-α-2 cells with knockdown of LHX2. Cell viability was determined at 0, 24, 48, 72 h after siRNAs (si-LHX2-1 and si-LHX2-2) transfection. **C.** Images of clonogenic analysis for cell proliferation in A549 and LTEP-α-2 cells with knockdown of LHX2. **D.** Bar charts indicating clonogenic growth of A549 and LTEP-α-2 cells with knockdown of LHX2. *P < 0.05; **P < 0.01; ***P < 0.001.

To elucidate the role of LHX2 in NSCLC, we knockdowned LHX2 expression in A549 and LTEP-α-2 cells (Figure 5A) by two specific siRNAs, and examined whether knockdown of LHX2 can inhibit NSCLC cell proliferation. CCK-8 and clonogenic assays indicated that the proliferation of LHX2-silenced cells was significantly weakened when compared with that of control cells (Figure 5B, 5C and 5D). The results demonstrated that LHX2 can promote NSCLC cell proliferation.

### Knockdown of LHX2 inhibits cell cycle in NSCLC cells

**Figure 6:**
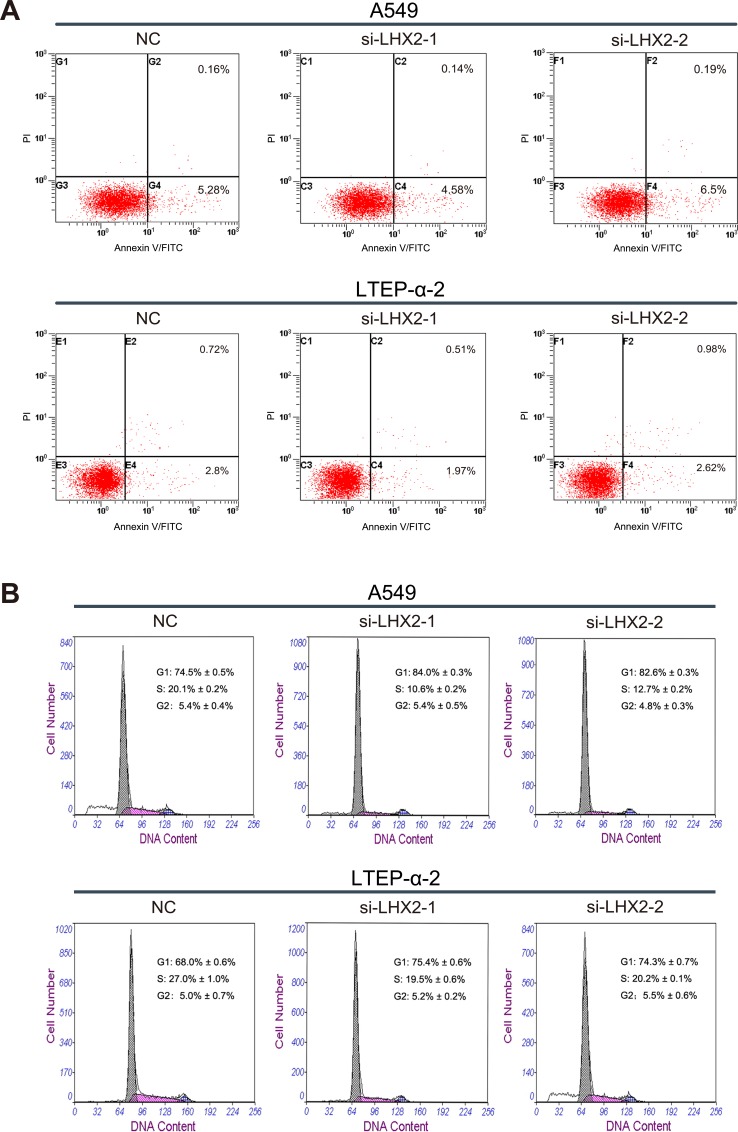
Knockdown of *LHX2* had no effect on cell apoptosis, whereas inhibits NSCLC cell cycle **A.** Flow cytometry apoptosis assay of A549 and LTEP-α-2 cells transfected with si-LHX2-1, si-LHX2-2 and nontargeting control siRNA (NC). Cells were harvested at 72 h post-transfection and stained with Annexin V/FITC and propidium iodide (PI). **B.** Flow cytometry cell cycle analysis of A549 and LTEP-α-2 cells transfected with si-LHX2-1, si-LHX2-2 and NC. Cells were harvested at 72 h post-transfection and stained with propidium iodide. Shown in the inset of each panel were percentages of cell cycle phases, in which values represent mean ± SD of three measurements.

To further investigate how LHX2 promotes NSCLC cell proliferation, we examined cell apoptosis and distribution of cell cycle phases in LHX2-silenced A549 and LTEP-α-2 cells. We found that transfection of si-LHX2 in A549 and LTEP-α-2 cells had no effect on cell apoptosis (Figure 6A), whereas LHX2 knockdown led to significant accumulation of cells in G1 phase (Figure 6B). Collectively, the results suggested that LHX2 promote cell proliferation by accelerating NSCLC cell cycle.

## DISCUSSION

LHX2 was originally identified as a transcriptional factor in pre-B cell lines and in the central nervous system [4, 20]. LHX2 plays crucial roles in many biological processes, such as embryogenesis [5], development [21] and cell differentiation [22]. Besides its roles in physiological conditions, LHX2 upregulation has been implicated in several types of human cancer [6]. However, the expression and regulation of *LHX2* in NSCLC has still not been elucidated.

To address this interesting question, we first compared the difference in *LHX2* expression between NSCLC cells and HBE cells. Elevated expression of LHX2 was found in NSCLC cells, supporting the findings that LHX2 serves as a tumor promoter in breast cancer cells [6]. Similarly, *LHX2* expression was up-regulated in NSCLC tissues. Moreover, our findings suggested that siRNA-induced knockdown of LHX2 significantly inhibited NSCLC cell proliferation by repressing NSCLC cell cycle. This is comparable with the findings in human pancreatic adenocarcinoma cells [23]. Therefore, our findings demonstrated that LHX2 may play a tumor-promoting role in NSCLCs.

Although there are more and more evidence showing that LHX2 is frequently up-regulated in human cancers [6-8, 23], the mechanisms underlying the increased expression of LHX2 are poorly understood. In this study, we focused on NSCLC cells and tissues to determine whether miRNAs can epigenetically influence *LHX2* expression. Intriguingly, miR-1238 level was reduced in NSCLC cells and tissues, which was significantly inversely correlated with *LHX2* expression. Of note, 77.4% of NSCLC tissues with low expression of miR-1238 displayed high expression of *LHX2* mRNA. The results imply that miR-1238 functionally contributes to the expression of *LHX2* in NSCLCs. In fact, this not surprising because miRNAs have been generally recognized to regulate gene expression at post-transcriptional level by targeting mRNAs [24]. Although several miRNAs including miR-1238 were reported to be implicated in colon cancer, the targets of miR-1238 has not yet been identified [25]. Thus, we performed an *in silico* prediction of microRNA targets and identified that miR-1238 can potentially bind to two target sites of *LHX2* 3′-UTR. Next, we adopted two methods to confirm whether *LHX2* is a *bona fide* target of miR-1238. Firstly, a luciferase reporter assay showed that miR-1238 can selectively target one putative site of *LHX2* 3′-UTR. Secondly, overexpression of miR-1238 in NSCLC cells significantly inhibited LHX2 mRNA and protein expression. This provides a strong rationale for our findings that both low miR-1238 and high *LHX2* are expressed in NSCLCs.

Given the facts that miR-1238 is frequently down-regulated not only in human cancer tissues [17-18], but also in human epithelia-derived cancer cells [19, 26] and our data that LHX2 is required for NSCLC cell proliferation (Figure 5), leading us consider the possibility that miR-1238 may inhibit NSCLC cell proliferation. As expected, this is first evidence of identifying the cytostatic role of miR-1238 in NSCLC cells, further improving our understanding of the tumor-promoting function of LHX2. Of course, we can not exclude the opposite role of miR-1238 played in other types of cancer, because a single miRNA may have different functions depending on the cellular context [27]. In deed, Haj-Ahmad *et al*. also found that miR-1238 was over-expressed in 38% of the initial prostate cancer samples, and suspected that miR-1238 may target SASH1 (SAM & SH3 domain containing protein 1) [26] which was down-regulated in breast cancer [28]. This mechanism might partially help us explain the reason for up-regulated miR-1238 in 38.0% of primary NSCLC, albeit further studies are warranted to validate the interaction of miR-1238 and SASH1.

In conclusion, this is the first report that miR-1238 is down-regulated in NSCLC, which is reversely correlated with the expression of *LHX2*. Mechanistically, miR-1238 inhibits *LHX2* expression by directly targeting *LHX2* 3′-UTR, and thereby represses NSCLC cell proliferation. Our findings shed light on the mechanistic interaction of miR-1238 and *LHX2* in NSCLC carcinogenesis. MiR-1238-mediated downregulation of LHX2 provides new insight into the therapy strategy for NSCLC.

## MATERIALS AND METHODS

### Cell culture

Human NSCLC cells A549, LTEP-α-2 and H1299 (lung adenocarcinoma cell lines) and H460 (large cell lung cell line) from the Cell Bank of the Chinese Academy of Sciences (Shanghai, China) and human bronchial epithelial (HBE) cells (Bogoo Biotechnology, Shanghai, China) were cultured in RPMI 1640 medium (HyClone, South Logan, UT, USA) with 10% FBS, L-glutamine and antibiotics (Invitrogen, Carlsbad, CA, USA) and incubated at 37°C in 5% CO_2_.

### Human NSCLC tissue samples

Fifty paired NSCLC tissues and adjacent noncancerous lung tissues were obtained after informed consent from patients in the First Affiliated Hospital of Soochow University. Histological and pathological diagnostics for NSCLC patients were evaluated according to the Revised International System for Staging Lung Cancer. The demographic and clinical characteristics were described for each patient with NSCLC (Supporting Information Table S1). None of the patients received any anticancer therapy before tissue sampling. Tissue samples were snap-frozen and stored in a deep-freezer at −80°C until analyzed. This study was approved to be performed by the Academic Advisory Board of Soochow University.

### RNA extraction, cDNA synthesis, and real-time quantitative reverse transcriptase-polymerase chain reaction (qRT-PCR)

Total RNA was isolated from cells and tissues using a HP Total RNA Kit (Omega Biotech, Stamford, CT, USA) according to the manufacturer's protocol. Synthesis of cDNA with reverse transcriptase was performed using an M-MLV First Strand Kit (Life Technologies, Gaithersburg, MD, USA). Real-time qRT-PCR was conducted on an ABI Prism 7500 Real-Time PCR system (Applied Biosystems, Foster City, CA, USA) using the SYBR-Green-based method. Primer sequences for *LHX2* mRNA, miR-1238, β-actin and U6 detection are described in Table 1. Relative expression levels of *LHX2* mRNA and miR-1238 were determined following normalization to β-actin and U6, respectively.

**Table 1:** Primers for reverse transcription or amplification of the mature miR-1238 and U6, LHX2 mRNA and β-actin

Name	Sequence (5′ - 3′)
RT Primers	
miR-1238	GTCGTATCCAGTGCAGGGTCCGAGGTATTCGCACTGGATACGACGGGGCAGA
U6	CGAGCACAGAATCGCTTCACGAATTTGCGTGTCAT
qRT-PCR Primers	
miR-1238	F: GTCGTATCCAGTGCAGGG; R: CGACGCTTCCTCGTCTG
U6	F: CGAGCACAGAATCGCTTCA; R: CTCGCTTCGGCAGCACATAT
LHX2 mRNA	F: TTCCAGAACGCCCGAGCCAA; R: GGGGCTAGTCAAGTCTGTC
β-actin	F: CACAGAGCCTCGCCTTTGCC; R: ACCCATGCCCACCATCACG

F, forward; R, reverse.

### Western blot analysis

Protein products from cell lysates were fractionated by 10% SDS-PAGE electrophoresis and transferred to nitrocellulose membranes (Millipore, Billerica, MA, USA). Membranes were blocked with BSA/TBST buffer for 1 h and then incubated with primary antibodies overnight at 4°C followed by incubation with HRP-conjugated secondary antibodies. Detection was performed using ECL kit (Pierce, Rockford, IL, USA). Each experiment was done in triplicate. LHX2 expression was normalized against β-actin. Antibodies used in the analysis were as follows: anti-LHX2 and anti-β-actin (Santa Cruz Biotechnology, Santa Cruz, CA, USA), and anti-rabbit and -mouse secondary antibodies (Santa Cruz Biotechnology).

### Construction of luciferase reporter plasmid, transient transfection and luciferase assay

A 219-bp fragment of human *LHX2* 3′-UTR containing two predicted miR-1238 target sites (positions 176-182 and 244-251) was amplified using the following primers: forward, 5′-CCGCTCGAGGAGCAACTAACTAACCACA-3′ (*Xho*I); reverse, 5′-ATTTGCGGCCGCCGTGGCAGTCTTTGAAAAT-3′ (*Not*I), and subcloned into a psiCHECK-2 vector with restriction enzymes *Xho*I and *Not*I (underscored; Fermentas, Hanover, MD, USA) to create a psiCHECK-2-*LHX2*-3′-UTR-wildtype. Comparably, 7 or 14 mutational bases were introduced into the two predicted miR-1238 target sites to obtain three mutated fragments. The three mutated fragments were directly synthesized (Sangon Biotech, Shanghai, China) and subcloned into the psi-CHECK-2 vector to generate the respective psiCHECK-2-*LHX2*-3′-UTR-mutant. Then, 50 ng of the above-described luciferase constructs were cotransfected into cells with 20 nM of either miR-1238 mimic (5′-CUUCCUCGUCUGUCUGCCCC-3′) or negative control (NC), respectively. A scrambled sequence (5′-UUCUCCGAACGUGUCACGUTT-3′) was used as NC. All the transient transfections were done using Lipofectamine 2000 (Invitrogen). 48 hours later, cells were harvested and analyzed for luciferase activities using the Dual-Luciferase Reporter Assay System (Promega, Madison, WI, USA). Each experiment was performed in triplicate. Results are represented as relative *Renilla* luciferase activities, which are obtained following normalization to *firefly* luciferase activities.

### Transient RNA interference

Two pre-designed short interfering RNA (siRNA) sequences, which target human *LHX2*, were directly synthesized (GenePharma, Shanghai, China). Sequences for LHX2 siRNAs are as follows: si-LHX2-1, 5′-GCTTCGGACCATGAAGTCTTA-3′; si-LHX2-2, 5′-GCAACCTCTTACGGCAGGAAA-3′. A scrambled sequence (5′-TTCTCCGAACGTGTCACGT-3′) was used as nontargeting control siRNA (NC). Cells were transiently transfected with 100 pmol of siRNA sequences using Lipofectamine 2000 (Invitrogen). After 72 h transfection, the cells were collected for further experiments.

### Cell viability assay

Cell viability was assessed by the Cell Counting Kit-8 (CCK-8) kit (Dojindo Laboratory, Kumamoto, Japan) according to the manufacturer's instructions. Briefly, 1,000∼2,000 cells transfected with miR-1238 mimics and si-LHX2 or NC were seeded into each well of a 96-well plate. After incubation, 10 μl of CCK-8 was added to 90 μl culture media. Subsequently, the cells were incubated for 1 h at 37°C and the plates were analyzed on an MRX Microplate Reader (Dynex Technologies, West Sussex, UK) at 450 nm to measure the absorbance. The experiment was repeated at least three times.

### Cell proliferation assay

Cell proliferation was determined using clonogenic assay. Briefly, cells transfected with miR-1238 mimics and si-LHX2 or NC were diluted in complete culture medium with a grad of 200 cells and reseeded in a 60 mm plate. After incubation for 14-20 days, depending on cell growth rate, foci formed by least 50 cells were stained with Giemsa and counted.

### Cell cycle analysis

Propidium iodide (PI) staining flow cytometry was used to assess distribution of cell cycle phases. According to Cell Cycle Analysis Kit (Beyotime, Shanghai, China), Cells were transfected with si-LHX2-1, si-LHX2-2 and NC for 72 h in 6-well plates. Then cells were harvested and fixed in 70% ethanol at 4°C overnight and stained in a mixture of PI/RNase A. Finally, after being kept in the dark at 37°C for 30 min, the staining cells were analyzed using a flow cytometer (FC500 Flow Cytometer; Beckman Coulter, USA).

### Cell apoptosis assay

Cells were transfected with si-LHX2-1, si-LHX2-2 and NC in 6-well plates. After 72 h, cells were harvested, rinsed with PBS and resuspended in 195 μl 1X binding buffer containing 5 μl Annexin V/FITC and 10 μl propidium iodide (Annexin V/FITC kit; Beyotime). Then cells were incubated at room temperature for 10∼20 min in the dark. The fluorescence of the cells was immediately measured using a flow cytometer.

### Statistical analysis

Differences in *LHX2* mRNA and miR-1238 expression between NSCLC tissues (T) and adjacent noncancerous lung tissues (N) were assessed by a paired *t* test (2-tailed). With respect to cell lines, differences between two groups were evaluated using an unpaired *t* test (2-tailed). Correlation between expression level ratios (T/N) of *LHX2* mRNA and miR-1238 was analyzed using the Spearman rank correlation test. Data are presented as means ± SE. Two-way ANOVA was used to compare the difference in cell proliferation between 2 groups. All statistical analyses were performed using GraphPad Prism 5.02 software (GraphPad, San Di-ego, CA, USA) and SPSS 16.0 software (SPSS, Chicago, IL, USA), and a p-value of < 0.05 was considered statistically significant.

## SUPPLEMENTARY TABLES


