# Supplementary material for: miR-1238 inhibits cell proliferation by targeting LHX2 in non-small cell lung cancer
Source: Oncotarget. 2015 May 22;6(22):19043–54. doi: 10.18632/oncotarget.4232 (PMC4662474; doi:10.18632/oncotarget.4232)
Supplement: Supplementary file 1 [file oncotarget-06-19043-s001.pdf]

miR-1238 inhibits cell proliferation by targeting LHX2 in non-small cell lung cancer

Supplementary Material

Supplemental Table S1: Demographic and clinical characteristics of 50 NSCLC patients and relative expression of LHX2 mRNA and miR-1238 in 50 paired NSCLC tissues

Case	Age (years)	Histology *	TNM	Stage	LHX2 mRNA (T/N) †	miR-1238 (T/N) †
1	68	SqC	T2N2M0	IIIA	↑	↓
3	39	AdC	T1N0M0	IA	↑	↓
4	60	SqC	T1N0M1	IV	↑	↓
5	54	SqC	T4N1M0	IIIA	↑	↓
6	69	SqC	T4N2M1	IV	↓	↓
7	79	SqC	T2N0M0	IB	↑	↑
8	71	SqC	T2N0M0	IIA	↑	↓
9	55	SqC	T4N1M1	IV	↑	↓
10	70	AdC	T4N0M1	IV	↓	↑
11	53	AdC	T2N0M0	IB	↓	↑
12	55	SqC	T3N1M0	IIIA	↓	↑
13	64	AdC	T1N0M0	IA	↑	↓
15	58	Other	T4N0M0	IIIA	↑	↑
17	77	Other	T3N0M0	IIB	↓	↓
18	74	SqC	T3N1M0	IIIA	↓	↑
19	67	AdC	T2N1M0	IIB	↑	↓
22	69	AdC	T2N0M1	IV	↓	↓
23	61	Other	T2N0M0	IB	↑	↑
24	60	SqC	T4N1M0	IIIA	↓	↑
25	72	AdC	T1N0M0	IA	↑	↑
26	70	AdC	T1N0M0	IA	↓	↑
30	68	AdC	T2N0M0	IB	↑	↓
31	58	Other	T3N0M0	IIB	↓	↑
33	65	AdC	T2N1M0	IIA	↑	↓
34	70	SqC	T2N0M1	IV	↑	↓
35	73	SqC	T3N0M0	IIB	↑	↓
36	49	SqC	T3N1M0	IIIA	↑	↑
40	60	Other	T1N2M0	IIIA	↑	↓
48	78	AdC	T1N0M0	IA	↑	↓
49	67	AdC	T2N0M0	IIA	↓	↓
50	68	SqC	T3N0M0	IIB	↓	↑
51	68	AdC	T2N0M0	IB	↑	↓
52	67	AdC	T2N0M0	IB	↑	↓
53	70	AdC	T1N0M0	IA	↓	↓
54	62	AdC	T2N0M0	IB	↓	↓
56	69	SqC	T2N0M0	IIA	↑	↑
57	73	AdC	T2N0M0	IB	↑	↓

58	52	AdC	T2N2M0	IIIA	↑	↓
59	79	AdC	T2N0M0	IIA	↑	↓
60	69	AdC	T2N0M0	IIA	↑	↓
61	72	Other	T2N0M0	IB	↑	↓
62	78	AdC	T2N0M0	IIA	↑	↑
63	61	Other	T2N2M0	IIIA	↑	↑
64	68	AdC	T2N0M0	IIA	↓	↓
65	73	Other	T2N2M0	IIIA	↑	↓
66	68	AdC	T1N0M0	IA	↑	↓
67	64	AdC	T1N2M0	IIIA	↑	↑
68	63	AdC	T1N0M0	IA	↑	↑
69	29	AdC	T2N0M1	IV	↑	↓
71	69	SqC	T2N0M0	IIA	↑	↑

* AdC, adenocarcinoma; SqC, squamous cell carcinoma; Other, large cell carcinoma, etc.

† ↑, increased (T/N > 1.0); ↓, reduced (T/N < 1.0); T, NSCLC tissues; N, paired noncancerous lung tissues.

Supplemental Table S2: Comparison between various clinicopathologic parameters and levels of LHX2 mRNA and miR-1238 expression in NSCLC tissues

Parameter	n	LHX2 mRNA	miR-1238
Age			
>65	30	0.001767±0.000333	0.001011±0.000592
<65	20	0.003080±0.001019	0.000509±0.000180
P value		0.1607	0.5026
Smoking status			
Yes	31	0.002057±0.000469	0.000272±0.000077
No	19	0.001416±0.000932	0.000521±0.000175
P value		0.4985	0.1484
Histology			
Adenocarcinoma	26	0.002187±0.000635	0.000896±0.000633
Squamous cell carcinoma	16	0.002372±0.000729	0.000790±0.000482
Others	8	0.002244±0.000110	0.000570±0.000250
P value		0.9835	0.9527
Clinical stage			
I	17	0.003198±0.001082	0.001383±0.000906
II	14	0.000805±0.000224	0.000187±0.000076
III	12	0.002321±0.000830	0.000923±0.000645
IV	7	0.002651±0.000416	0.000274±0.000111
P value		0.2096	0.5643
